# Characterizing carbonate mineral-forming bacterial strains and their carbonic anhydrase activities in two coastal sabkhas

**DOI:** 10.1016/j.bbrep.2025.102064

**Published:** 2025-05-27

**Authors:** Khaled Naja, Sara H. Alhadidi, Hadil Elsayed, Jassim Abdulla A. Al-Khayat, Fadhil Sadooni, Hamad Al Saad Al-Kuwari, Zulfa Ali Al Disi

**Affiliations:** a Environmental Science Centre, Qatar University, P.O. Box 2713, Doha, Qatar; b Biomedical Research Centre, QU health, Qatar University, Doha, Qatar

**Keywords:** Carbonic anhydrase, Biomineralization, *Virgibacillus*, Carbonate minerals, Sabkha

## Abstract

The enzyme carbonic anhydrase (CA) plays a key role in carbonate mineral formation by facilitating the interconversion between CO_2_ and bicarbonate ions, thus influencing carbonate precipitation processes in natural environments. This study investigates the biomineralization potential of *Virgibacillus* strains isolated from two distinct coastal sabkhas in Qatar—Dohat Faishakh Sabkha (DFS) and Khor Al-Adaid Sabkha (KAS)—to better understand the enzymatic mechanisms driving carbonate formation in hypersaline environments. The isolated strains were evaluated for mineral formation and CA activity using three artificial media designed to simulate natural conditions: MD1, seawater-based with tryptone (SWTr), and evaporated seawater-based with tryptone (EWTr). While all strains demonstrated the ability to form minerals in MD1, only *Virgibacillus salarius* and *Virgibacillus marismortui*, both exclusive to DFS, exhibited robust mineral precipitation in SWTr and EWTr media. These strains also showed significantly higher CA activity compared to *Virgibacillus chiguensis* and *Virgibacillus dokdonensis*, which were present in both sabkhas but displayed limited mineralization and low enzymatic activity under saline conditions.

Statistical analyses, including ANOVA and principal component analysis (PCA), confirmed the significant role of CA activity and salinity in modulating biomineralization potential among these strains. This research underscores the potential of CA-driven biomineralization for environmental applications. The ability of *V. salarius* and *V. marismortui* to precipitate carbonates under high-salinity conditions positions them as promising candidates for bio-based carbon sequestration technologies.

## Highlights

• Carbonic anhydrase activity is strongly associated with carbonate mineral formation under varying environmental conditions.
• All strains formed minerals in MD1, whereas only *V*. *salarius* and *V*. *marismortui* formed minerals in seawater-based media.
• *V*. *salarius* and *V*. *marismortui* exhibited high enzymatic activity and significant mineral formation potential in saline media.
• *V*. *chiguensis* and *V*. *dokdonensis* demonstrated limited mineral formation and low enzymatic activity in seawater-based media.

## 1 Introduction

The formation of carbonate minerals is a fundamental natural process significantly influenced by biological activity [1,2]. In recent years, biomineralization by aerobic mineral-forming bacteria has garnered significant attention, especially within extreme environments such as sabkhas—salt flats characterized by high salinity and unique geochemical properties—because these bacteria play a crucial role in mediating carbonate precipitation, influencing sedimentary processes, and potentially contributing to early diagenesis. [[3], [4], [5]]. These bacteria play a key role in mediating the precipitation of carbonate minerals, primarily through their metabolic activities [6,7].

Two key enzymes involved in bacterial biomineralization are carbonic anhydrase (CA) and urease, which function through distinct mechanisms [8,9]. CA catalyzes the reversible conversion of carbon dioxide (CO_2_) and water into bicarbonate ions (HCO_3_^−^) and protons (H^+^), providing a source of bicarbonate essential for calcium carbonate (CaCO_3_) precipitation [10,11]. This process helps maintain the supersaturation conditions required for mineral formation, particularly in environments with high CO_2_ levels, such as marine settings [12]. In contrast, urease catalyzes the hydrolysis of urea into ammonia (NH_3_) and CO_2_, leading to the production of hydroxide ions (OH^−^) that raise the pH of the surrounding environment [13]. This increase in alkalinity promotes the rapid precipitation of CaCO_3_, making urease-mediated biomineralization more efficient in environments where urea is present [14].

The occurrence and functionality of mineral-forming bacteria in sabkhas are significantly influenced by the distinctive biogeochemical characteristics of these environments, which can vary widely due to factors such as salinity, organic matter content, and redox conditions [15,16]. For instance, sabkhas with distinct biogeochemical properties may support unique bacterial communities [17]. These communities, often dominated by extremophilic bacteria adapted to hypersaline and alkaline conditions, play a crucial role in local biogeochemical cycles by thriving under and responding to their respective environmental conditions [18,19]. Understanding the function of CA and other bacterial enzymes in mineral formation within such systems has broader implications for our knowledge of carbon cycling and sediment dynamics in extreme environments [20,21]. Moreover, leveraging these microbial processes can pave the way for environmentally sustainable technologies, including microbial-driven carbon capture and geoengineering applications aimed at mitigating climate change. Research has emphasized the importance of both aerobic and anaerobic pathways in the biomineralization processes of sabkha systems, highlighting the adaptive capabilities of microbial communities to thrive under diverse environmental conditions [[22], [23], [24], [25]]. These studies not only illuminate the adaptive strategies of bacteria in harsh environments but also underscore the broader impact of microbial activities on carbonate mineral formation and sedimentary processes [26,27]. Such insights are crucial for understanding the complexities of biogeochemical cycles, sediment formation, and the potential applications of these microbial processes in environmental management and biotechnological fields [28,29].

This study investigates the role of *Virgibacillus* strains isolated from Dohat Faishakh Sabkha (DFS) and Khor Al-Adaid Sabkha (KAS) in carbonate mineral formation, with a focus on the activity of CA. MD1, a nutrient-rich medium, along with SWTr and EWTr, which simulate seawater and sabkha water conditions, were used under controlled settings. The mineralization potential of these strains was examined under diverse conditions. The enzymatic activity of CA was compared between strains capable of forming minerals across all media and those forming minerals only under specific conditions. The findings provide insights into the metabolic and functional diversity of *Virgibacillus* strains and the environmental factors that influence their biomineralization capabilities.

## 2 Materials and methods

### 2.1 Study sites and sampling periods

**Fig. 1:**
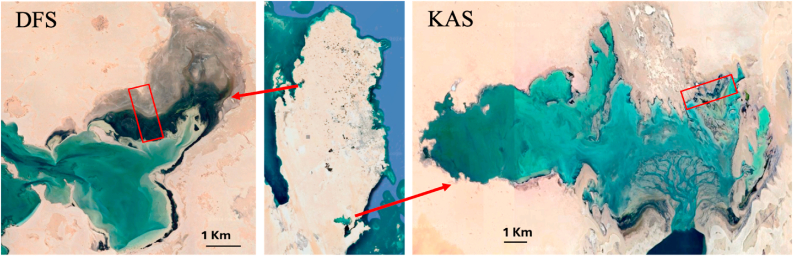
Map of Qatar showing sampling points in Dohat Faishakh Sabkha (DFS) and Khor Al Adaid Sabkha (KAS).

Core samples were collected seasonally in February and October from 2022 to 2024 at two distinct sabkhas in Qatar: KAS and DFS. KAS, located in southeastern Qatar, is characterized by its hypersaline conditions, whereas DFS, situated along the northwest coast of the Qatar Peninsula, is an evaporitic environment predominantly composed of carbonate minerals (Fig. 1). These contrasting environments provide an ideal framework for investigating the interplay between environmental factors and mineral formation.

For this study, samples collected in February and October were analyzed to capture the seasonal variability in carbon stocks and environmental conditions, as previously reported by Al Disi et al. [30]. These months were chosen as they effectively represent the seasonal extremes in carbon stock dynamics. DFS exhibited minimal variability, while KAS showed pronounced fluctuations, underscoring the contrasting ecological and geochemical processes in the two sabkhas.

### 2.2 Culture media

Modified urea agar (g/L) (5 NaCl, 2 KH_2_PO_4_, 1 glucose, 0.012 phenol red indicator, 0.2 peptone, 30 agar, and 20 filtered urea), with a final pH of 6.8, as described by Bibi et al. [31], was used for screening ureolytic bacteria. MD1 medium was used to investigate the mineral-forming potential of the studied strains [null]. Additionally, two other artificial media—SWTr and EWTr—were prepared using artificial seawater and evaporated seawater, respectively. These media were designed to mimic natural environments. The compositions of these media were developed based on extensive preliminary research and are detailed in Table 1. All media were sterilized by autoclaving at 121 °C for 20 min, and the pH was adjusted to 7.0 using 1 M KOH prior to sterilization.

**Table 1:** Composition of MD1, artificial seawater-based, and evaporated seawater-based media used in the study.

Media/Component (g/L)	MD1	SWTr	EWTr
Yeast Extract	10	–	–
Tryptone	5	5	5
NaCl	35	25	93
Mg-Acetates	12	–	–
Ca-Acetates	1.5	–	–
MgCl_2_	–	10.7	35.3
CaCl_2_	–	1.5	4.6
KCl	–	0.7	2.6
Na_2_SO_4_	–	3.9	13.6
KBr	–	0.1	0.3

### 2.3 Isolation and purification of the bacterial strains

Enrichment cultures were prepared by suspending 2 g of sediments in 20 mL of liquid MD1 medium. The cultures were incubated for 72 h in a shaker at 30 °C and 200 rpm. Subsequently, serial dilutions were performed for each culture, and 100 μL of each dilution was spread onto MD1 solid medium plates. Light microscopy was used to examine the bacterial cells from each colony, along with observations of colony morphology and color. The most representative colonies were selected from those formed at the highest dilution level, 10^−4^. Purification was performed using the streak plate method to ensure that each plate contained a single, isolated strain.

### 2.4 Molecular identification of the bacterial strains

Total DNA was extracted from different bacterial strains exhibiting experimental mineral formation using the DNeasy® PowerBiofilm® Kit (Qiagen, Germany) according to the kit protocol. The quality of each DNA sample was evaluated using 1 % (w/v) agarose gel electrophoresis and quantified by spectrophotometry on a Nanodrop. Subsequently, 1 μL of each DNA sample was used as a template in a PCR reaction with a total volume of 25 μL that consisted of 0.5 mM of the pair universal primers (RibS74sp and RibS73sp, [4]), 12.5 μL master mix (DreamTaq™ PCR Master Mix (2x), Thermo Scientific, Vilnius-Lithuania), and nuclease-free water up to 25 μL. The amplification reactions were carried out using the following amplification program: 3 min at 94 °C, followed by 35 cycles of 30 s at 94 °C, 30 s at 60 °C, and 30 s at 72 °C, with a final extension step at 72 °C for 2 min. Afterward, the DNA amplicons were purified with the PCR purification kit (Gene JET PCR, Thermo Scientific, USA) as per the protocol provided by the manufacturer. Sanger sequencing was conducted in the laboratories of Weill Cornell Medicine-Qatar. The nucleotide sequences were analyzed with BLAST at the NCBI database. The phylogenetic tree of bacterial isolates was generated using MEGA X software, employing the maximum likelihood (ML) method based on 16S rDNA gene sequences.

### 2.5 Screening for urease activity

Urea Media culture plates were inoculated with bacterial strains and incubated in the dark at 30 °C. Bacterial growth, accompanied by a change in color to pinkish-red, indicated an increase in pH, signifying ureolytic activity.

### 2.6 Evaluation of the mineral formation by the isolated bacterial strains

MD1, SWTr, and EWTr solid media were used to assess the mineral-forming potential of the isolates, following the method described by Al Disi et al. [3]. Each isolate was inoculated onto the solid media and incubated at 30 °C for three weeks. The inoculated plates were periodically examined under a light microscope to observe crystal formation. The formed minerals were recovered from pure cultures of the mineral-forming strains as described by Al Disi et al. [3]. SEM images were obtained using a Nova Nano Scanning Electron Microscope equipped with a Bruker EDS Detector with 5 nm resolution and a magnification of 200,000 × . The bulk mineralogical composition of the recovered minerals was determined using a PANalytical multipurpose Empyrean X-ray diffractometer.

### 2.7 Determination of carbonic anhydrase enzymatic activity

#### 2.7.1 Bacterial culture and Sample preparation

Culture plates were inoculated with bacterial cells from each strain on MD1, SWTr, or EWTr agar media. The plates were then incubated at 30 °C for 3 days, as the highest CA activity was previously recorded at this time point by Abdelsamad et al. [34]. After the incubation period, 1 μL of culture was collected using a calibrated 1 μL loop and suspended in 200 μL of ice-cold Tris-Saline Buffer (TBS) in a sterile Eppendorf tube. The suspension was centrifuged at 3000×*g* for 5 min, and the resulting pellet was washed twice with 200 μL of ice-cold TBS. Subsequently, the washed pellet was resuspended in 150 μL of ice-cold lysis buffer and kept on ice for 10 min. Complete cell lysis was ensured by freezing the samples at −80 °C for 15 min.

#### 2.7.2 Esterase activity assay

The esterase activity assay was conducted following the method outlined by Ramanan et al. (2009). For the assay, 10 μL of the prepared sample was mixed with 85 μL of assay buffer. Then, 10 μL of the supernatant was added (in duplicate) into designated wells of a 96-well plate, and the total volume of each well was adjusted to 95 μL using CA assay buffer. The mixture was thoroughly mixed, and 5 μL of *p*-NPA substrate solution was added to each well to initiate the reaction.

Absorbance at 405 nm was measured using a microplate reader (FLUOstar Omega) over a 30-min period. A standard curve, prepared with varying concentrations of *p*-NPA, was used to calculate the production of the yellow product, *p*-nitrophenol, based on optical density. The *p*-NPA stock solution was freshly prepared by dissolving 54 mg of *p*-NPA in 5 mL of ethanol with vigorous agitation. The stock solution was stored at 4 °C and used within 1 h. For experimental use, 1.5 mL of the stock solution was diluted with 8.5 mL of distilled water.

All measurements were performed in triplicate, and the results are reported as the mean ± standard deviation of the obtained values.

One unit of esterase activity is defined as the amount of enzyme required to produce 1 μmol of *p*-nitrophenol per minute at room temperature. One unit of CA activity is the amount of enzyme that catalyzes the release of 1 μmol of nitrophenol per minute from the substrate under the assay conditions at 25 °C. The CA activity was calculated according to Equation (1).

Equation (1) $$\text{CA}\;\text{activity}=\frac{B\;x\;D\;x\;1000}{△T\;x\;V}=mU/ml$$

B = Released Nitrophenol in sample based on standard curve slope (nmol).

D = Dilution Factor (D = 1 when Samples are undiluted)

1000 = 1 mL ≡ 1000 μL

ΔT = Reaction time (minutes)

V = Sample volume (μL)

While the esterase assay using *p*-nitrophenyl acetate (*p*-NPA) is not specific to carbonic anhydrase (CA), it offers a rapid and scalable approach suitable for multi-strain screening. We tested the Wilbur-Anderson assay on selected samples, but the results were highly variable and inconsistent across replicates, likely due to its low sensitivity and subjective endpoint detection. Given these limitations, the esterase assay was deemed more appropriate for comparative analysis in this study. Partial inhibition with acetazolamide supported CA involvement in the esterase activity measured.

## 3 Results

### 3.1 Isolation and identification of the bacterial strains isolated from sabkha sediments

Bacterial strains were isolated from two coastal sabkhas, KAS and DFS, during multiple collection periods spanning from February 2022 to February 2024 (Table 2). presents a representative list of these strains, indicating their distribution across different sampling dates and locations.

**Table 2:** Representative list of the strains isolated from KAS and DFS during different collection periods.

Date	KAS		DFS	
Feb-22	KAS1	*Virgibacillus chiguensis*	DFS1	*Virgibacillus salarius*
Feb-22	KAS2	*Salinivibrio proteolyticus*	DFS2	*Salinivibrio costicola*
Feb-22	KAS3	*Virgibacillus dokdonensis*	DFS3	*Virgibacillus marismortui*
Oct-22	KAS4	*Virgibacillus dokdonensis*	DFS4	*Bacillus mojavensis*
Oct-22	KAS5	*Virgibacillus dokdonensis*	DFS5	*Bacillus vallismortis*
Oct-22	KAS6	*Salinivibrio costicola*	DFS6	*Bacillus vallismortis*
Oct-22	KAS7	*Salinivibrio costicola*	DFS7	*Virgibacillus chiguensis*
Oct-22	KAS8	*Virgibacillus dokdonensis*	DFS8	*Virgibacillus dokdonensis*
Oct-23	KAS9	*Virgibacillus chiguensis*	DFS9	*Bacillus haynesii*
Oct-23	KAS10	*Virgibacillus chiguensis*	DFS10	*Bacillus licheniformis*
Oct-23	KAS11	*Virgibacillus chiguensis*	DFS11	*Staphylococcus epidermidis*
Oct-23	KAS12	*Virgibacillus dokdonensis*	DFS12	*Virgibacillus chiguensis*
Oct-23	KAS13	*Virgibacillus dokdonensis*	DFS13	*Virgibacillus salarius*
Oct-23	KAS14	*Virgibacillus dokdonensis*	DFS14	*Virgibacillus salarius*
Feb-24	KAS15	*Virgibacillus chiguensis*	DFS15	*Virgibacillus chiguensis*
Feb-24	KAS16	*Virgibacillus dokdonensis*	DFS16	*Virgibacillus dokdonensis*
Feb-24	KAS17	*Virgibacillus dokdonensis*	DFS17	*Virgibacillus marismortui*
Feb-24	KAS18	*Staphylococcus pasteuri*	DFS18	*Virgibacillus salarius*
Feb-24	KAS19	*Bacillus licheniformis*	DFS19	*Virgibacillus salarius*

The isolated bacteria from the KAS and DFS sabkhas exhibit remarkable diversity, reflecting the adaptability of microbial communities to their distinct environmental conditions. Prominent genera include *Virgibacillus*, *Bacillus*, *Staphylococcus*, and *Salinivibrio*, with species such as *Virgibacillus dokdonensis* and *Virgibacillus chiguensis* consistently found across different sampling periods, highlighting their resilience in hypersaline environments. Notably, *Virgibacillus marismortui* and *Virgibacillus salarius* were exclusively isolated from the DFS sediments, which is characterized by a significantly different biogeological background, including higher salinity levels. This suggests that these species are uniquely adapted to thrive in more saline conditions. Meanwhile, other species, such as *Bacillus vallismortis* and *Staphylococcus epidermidis*, appeared sporadically, emphasizing the temporal variability of microbial composition. These findings underline the ecological complexity of sabkhas and the influence of salinity and other environmental factors in shaping their microbial communities. The strains of the *Virgibacillus* genus were selected to study their potential for mineral formation and to investigate their urease and CA activities.

### 3.2 Investigation of the mineral-forming potentials of the marine bacterial isolates

Table 3 summarizes the performance of various *Virgibacillus* strains in biomineralization processes, focusing on their urease activity, CA activity, and mineral formation capabilities under different environmental conditions. Mineral formation was evaluated based on growth and precipitation levels, with scores indicating no precipitation (−), moderate precipitation (+), or significantly higher precipitation (++).

**Table 3:** Investigation of the potentials of mineral formation by the bacterial strains.

Isolate ID	Strain name	Urease activity	CA Activity	Mineral formation		
				MD1	SWTr	EWTr
**DFS1**	*V*. *salarius*	NG	**+**	**+++**	**++**	**++**
**DFS3**	*V*. *martsimiure*	NG	**+**	**++**	**+**	**+**
**DFS8**	*V*. *salarius*	NG	**+**	**+++**	**++**	**++**
**DFS12**	*V*. *dokdonensis*	NG	**+**	**+**	–	–
**DFS15**	*V*. *chiguensis*	NG	**+**	**+**	–	–
**DFS17**	*V*. *martsimiure*	NG	**+**	**++**	**+**	**+**
**KAS1**	*V*. *dokdonensis*	NG	**+**	**+**	**-**	**-**
**KAS5**	*V*. *dokdonensis*	NG	**+**	**+**	**-**	**-**
**KAS11**	*V*. *chiguensis*	NG	**+**	**+**	**-**	**-**
**KAS16**	*V*. *chiguensis*	NG	**+**	**+**	**-**	**-**

Note: (NG) No visible growth (−) growth with no mineral formation. Qualitative Estimation of Crystals per mm^2^: +: 15–49 crystals/mm^2^, ++: 50–100 crystals/mm^2^, +++:>100 crystals/mm^2^.

The results demonstrate the diverse capabilities of *Virgibacillus* strains in biomineralization under varying environmental conditions. Notably, *V*. *salarius* (DFS8 and DFS1) exhibited superior mineral formation potential, achieving significantly higher precipitation (++), across all tested media (MD1, SWTr, and EWTr), highlighting its robustness and adaptability. Conversely, *Virgibacillus martsimiure* (DFS6 and DFS112) showed strong mineralization (++ in MD1 and SWTr), though with slightly reduced activity in the high-salinity EWTr medium. In contrast, *V*. *chiguensis* and *V*. *dokdonensis* strains displayed more limited capabilities, with only moderate precipitation (+) or no precipitation (−), suggesting that these species may be less effective in promoting mineral formation. The absence of urease activity across all strains suggests that CA activity is the primary enzymatic mechanism driving biomineralization, positioning these bacteria as promising candidates for applications in urea-independent environments. The results underscore the importance of medium composition, as MD1 and SWTr supported more robust precipitation, while the extreme salinity of EWTr presented a challenge for most strains, highlighting the need for further optimization of media to enhance mineral formation in harsh conditions.

### 3.3 Determination of the mineral composition

**Fig. 2:**
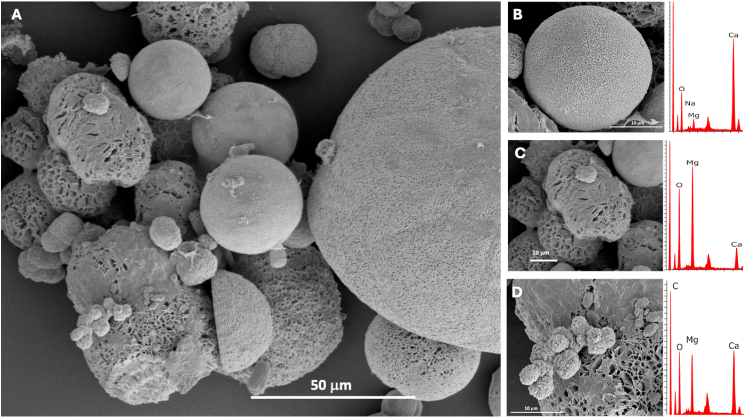
(A) Representative SEM image of minerals recovered from pure cultures of *V*. *salarius* DFS1 on MD1 medium. (B) Close-up SEM image with EDS analysis showing a calcium carbonate crystal. (C) Close-up SEM image with EDS analysis showing a magnesium carbonate crystal. (D) Close-up SEM image with EDS analysis showing a magnesium-calcium carbonate crystal.

**Fig. 3:**
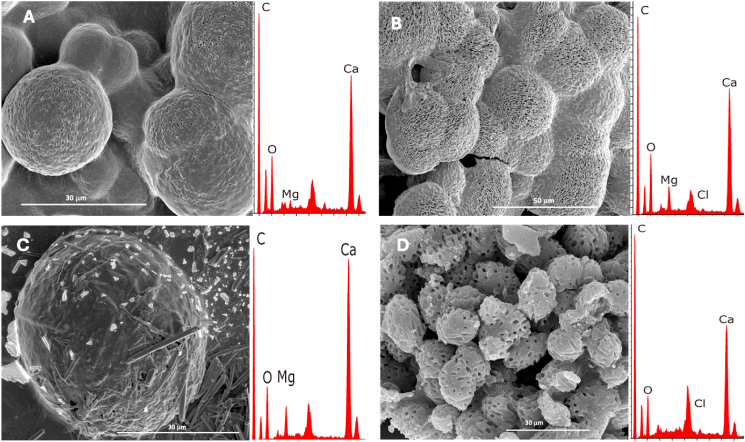
(A) SEM image of minerals recovered from pure cultures of *V. salarius* (DFS1) on SWTr, with EDS analysis indicating calcium carbonates. (B) SEM image of minerals recovered from *V*. *marismortui* (DFS3) on SWTr, showing calcium carbonates with low magnesium (Mg) content. (C) SEM image of minerals recovered from *V*. *salarius* (DFS1) on EWTr, with EDS analysis indicating calcium carbonates containing low Mg content. (D) SEM image of minerals recovered from *V*. *marismortui* (DFS3) on EWTr, showing calcium carbonates confirmed by EDS analysis.

The association between the growth of each mineral-forming bacterial strain and the composition of the formed crystals was determined using SEM/EDS analysis. Spherical-shaped crystals of varying sizes were formed by all the studied *Virgibacillus species* on MD1. The corresponding EDS indicates that they are composed of calcium carbonates, magnesium carbonate, and magnesium-calcium carbonates with varying Mg content (Fig. 2). In contrast, the minerals formed by *Virgibacillus salarius* (DFS1 & DFS8) and *Virgibacillus marismortui* (DFS3 & DFS17) on SWTr and EWTr were composed solely of calcium carbonate with low or negligible magnesium content (Fig. 3). No minerals were formed by *V*. *dokdonensis* and *V*. *chiguensis* in SWTr or EWTr.

**Fig. 4:**
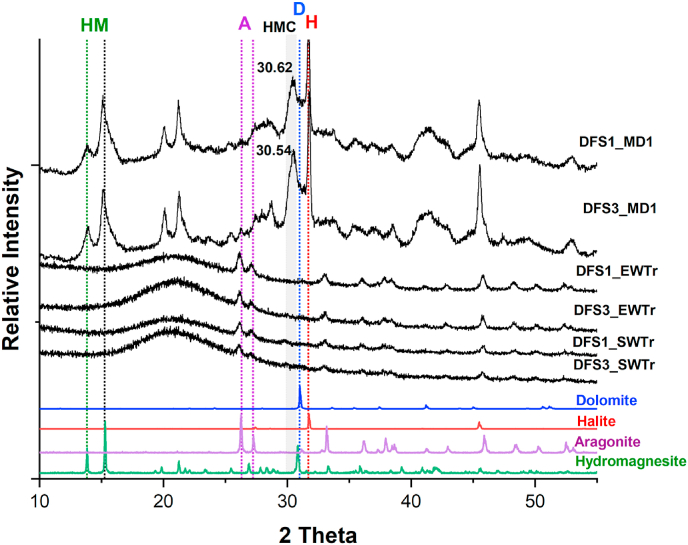
Comparisons of XRD patterns of minerals formed by *V*. *salarius* (DFS1) and *V. marismortui* (DFS3) strains grown on MD1, SWTr, and EWTr media. In MD1 cultures, the patterns show halite, hydromagnesite, calcite, and high-magnesium calcite (HMC) as indicated grey-highlighted panel. In contrast, only aragonite is observed in SWTr and EWTr cultures.

The XRD analysis confirmed the presence of hydromagnesite, calcite, and high-magnesium calcite in the minerals recovered from *V*. *salarius* and *V*. *marismortui* cultures grown on MD1 medium. In contrast, when these strains were grown on SWTr or EWTr, only aragonite was detected (Fig. 4).

In MD1 cultures, XRD analysis revealed a diffraction peak at 30.62° 2θ, which is indicative of high-magnesium calcite (HMC). This peak represents a shift from the standard calcite position (∼29.4° 2θ), consistent with partial substitution of Ca^2+^ by Mg^2+^ within the calcite lattice [21].

### 3.4 The role of carbonic anhydrase activity in carbonate mineral formation

**Fig. 5:**
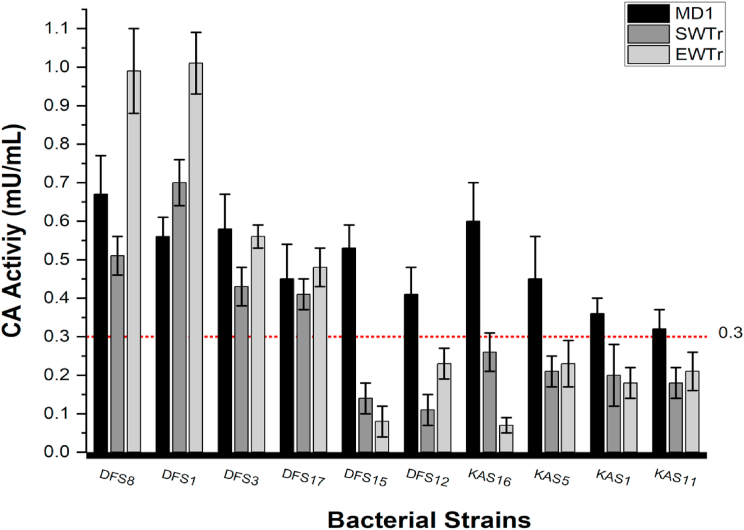
Intracellular esterase activities for the studied strains recorded after 72 h of incubation.

CA activity was determined for the studied strains under different growth conditions (Fig. 5). The CA activity of bacterial strains from DFS and KAS varied significantly across treatment conditions (MD1, SWTr, and EWTr). Most DFS strains exhibited higher CA activity in the evaporitic water treatment (EWTr), particularly DFS8 and DFS1, which showed the highest activity levels, exceeding 1.0 mU/mL. In contrast, the KAS strains demonstrated more consistent activity across all conditions, with some, such as KAS16 and KAS5, exceeding the 0.3 mU/mL threshold predominantly in the MD1 condition. *V*. *dokdonensis* and *V*. *chiguensis* from both sabkhas displayed consistently low CA activity across all treatments, remaining below the threshold. These results indicate that *V*. *salarius* and *V. marismortui* strains are more responsive to evaporitic water conditions, likely reflecting environmental adaptations, while KAS strains exhibit a more stable enzymatic profile, potentially linked to their hypersaline habitat.

Quantitative analysis revealed a strong linear correlation (R^2^ = 0.95, p < 0.00001) between carbonic anhydrase (CA) activity and mineral formation potential, as measured by crystal density score. This suggests that while qualitative CA presence (“+”) was observed across all strains, only those with CA activity above ∼0.6 mU/mL consistently produced dense carbonate precipitates (+++). These findings confirm that a threshold level of enzymatic activity is essential for effective biomineralization, reinforcing the need for quantitative rather than qualitative CA assessments in evaluating microbial mineralization capacity.

### 3.5 Statistical analysis

ANOVA results indicated a significant effect of media type on CA activity (p = 0.002), confirming variation in enzymatic expression across environmental conditions. Post-hoc Tukey's HSD tests showed that *V. salarius* (DFS1) had significantly higher CA activity than *V. dokdonensis* (KAS1, KAS5) and *V. chiguensis* (KAS11, KAS16) (p < 0.05).

**Fig. 6:**
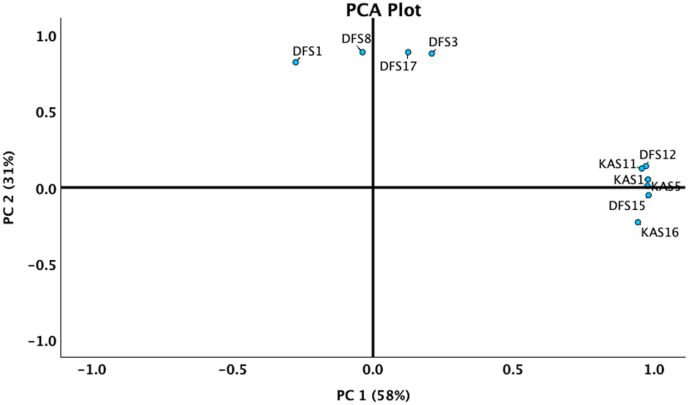
Principal Component Analysis (PCA) biplot illustrating the clustering of *Virgibacillus* strains based on CA activity and mineral formation potential.

Principal Component Analysis (PCA) was conducted to assess variation in CA activity and mineral formation potential among strains (see Fig. 6). PC1 explained 58 % of the variance, primarily associated with CA activity, while PC2 accounted for 31 % of the variance, distinguishing mineral-forming from non-mineral-forming strains. The PCA biplot clearly separated DFS and KAS strains, with *V. salarius* (DFS1, DFS8) and *V. marismortui* (DFS3, DFS17) clustering together due to high CA activity. Notably, *V. dokdonensis* (DFS12) and *V. chiguensis* (DFS15) clustered with KAS strains, suggesting potential metabolic or enzymatic adaptations.

## 4 Discussion

The observed differences in biomineralization potential among *Virgibacillus* strains suggest an influence of the distinct geochemical conditions of DFS and KAS. DFS, characterized by its evaporitic environment and salinities exceeding 300 %, is dominated by carbonate minerals such as gypsum, calcite, and dolomite [35,36]. These extreme conditions likely contribute to the stability of carbon stocks and the enhanced biomineralization potential of bacterial communities [37]. In contrast, KAS, characterized by siliciclastic sediments and microbial mats, exhibits greater mineralogical variability, with quartz as the dominant mineral and seasonal gypsum formation. Salinity in KAS fluctuates seasonally, ranging from 48 % to 140 % [30,36,38,39]. Additionally, DFS displays relatively stable carbon stocks and mineral composition across seasons, whereas KAS exhibits greater seasonal fluctuations in both, reflecting its more dynamic geochemical environment [30,40]. These environmental differences play a crucial role in shaping the metabolic and enzymatic adaptations of resident microbial populations [17].

The selection of *Virgibacillus* species for this study was based on their known ability to thrive in extreme conditions and their potential role in carbonate mineral formation [3].

A key observation was that none of the studied strains exhibited urease activity, indicating that carbonate precipitation in these environments is primarily mediated by CA activity rather than ureolysis. ANOVA results confirmed that both the type of media and the strain significantly influenced CA activity (p = 0.002), suggesting a role of the environmental conditions in enzymatic regulation. Post-hoc Tukey's HSD tests further showed that *V*. *salarius* (DFS1) exhibited significantly higher CA activity than *V*. *dokdonensis* (KAS1, KAS5) and *V*. *chiguensis* (KAS11, KAS16) (p < 0.05), emphasizing strain-specific differences in biomineralization potential. The selective precipitation of high-magnesium calcite (HMC) by *V. salarius* in MD1 medium implies that specific microbial mechanisms are actively influencing mineral formation pathways [32]. The production of extracellular polymeric substances (EPS), which are known to bind divalent cations such as Mg^2+^ may play a crucial role in these Mg-binding interactions within the EPS matrix. In addition, functional groups on the bacterial cell surface may serve as nucleation sites, guiding mineral orientation and promoting Mg incorporation into the calcite lattice [33,41,42]. These processes are consistent with early-stage dolomitization mechanisms observed in modern sabkha environments, where microbial mats create localized geochemical gradients that promote the formation of Mg-rich carbonates under ambient temperature conditions [25]. Thus, *V. salarius* may serve as a modern analog for microbial mediation of dolomite precursor phases in low-temperature evaporitic systems.

PCA was used to further resolve strain level differences based on CA activity and mineralization potential. PC1 explained 58 % of the variance, primarily driven by CA activity, while PC2 accounted for 31 % of the variance, distinguishing mineral forming from non-mineral forming strains. The PCA biplot revealed clear separation between DFS strains (*V. salarius* DFS1, DFS8 and *V. marismortui* DFS3, DFS17) and KAS strains (*V. dokdonensis* KAS1, KAS5 and *V. chiguensis* KAS11, KAS16), which aligns with the significant differences in CA activity observed in ANOVA. Additionally, the clustering of DFS12 and DFS15 with KAS strains in PCA further supports the idea that these strains may possess distinct enzymatic regulatory mechanisms compared to other 10.13039/100024533DFS strains.

Salinity emerged as a key factor influencing CA activity, but only in strains capable of mineral formation. For instance, the CA activity of *V. salarius* (DFS8) was recorded as 0.67 ± 0.1 mU/mL in MD1 (3.5 % NaCl), 0.51 ± 0.05 mU/mL in SWTr (2.5 % NaCl), and 0.99 ± 0.1 mU/mL in EWTr (9.3 % NaCl), demonstrating an adaptive enzymatic response to increasing salinity levels. In contrast, strains that failed to form minerals in SWTr or EWTr exhibited CA activities below 0.3 mU/mL, reinforcing the critical role of CA in biomineralization. These findings align with the ANOVA results, which showed a significant effect of media composition on CA activity, further confirming that nutrient availability and salinity stress influence CA regulation.

Quantitative analysis revealed a strong linear correlation (R^2^ = 0.95, p < 0.00001) between carbonic anhydrase (CA) activity and mineral formation potential, as measured by crystal density score. This suggests that while qualitative CA presence (“+”) was observed across all strains, only those with CA activity above ∼0.6 mU/mL consistently produced dense carbonate precipitates (+++). These findings confirm that a threshold level of enzymatic activity is essential for effective biomineralization, reinforcing the need for quantitative rather than qualitative CA assessments in evaluating microbial mineralization capacity.

The consistent performance of *V*. *salarius* and *V*. *marismortui* in mediating carbonate precipitation across various media suggests that specific metabolic and genetic adaptations enable these strains to thrive under diverse environmental conditions [5,43] Their elevated CA activity, particularly in the high-salinity EWTr medium, underscores their metabolic plasticity, facilitating carbonate formation in hypersaline environments. In contrast, *V*. *chiguensis* and *V*. *dokdonensis* exhibited limited mineralization capabilities, with carbonate precipitation restricted to the nutrient-rich MD1 medium. This limitation may be attributed to the absence or inefficiency of key pathways essential for biomineralization [19].

**Fig. 7:**
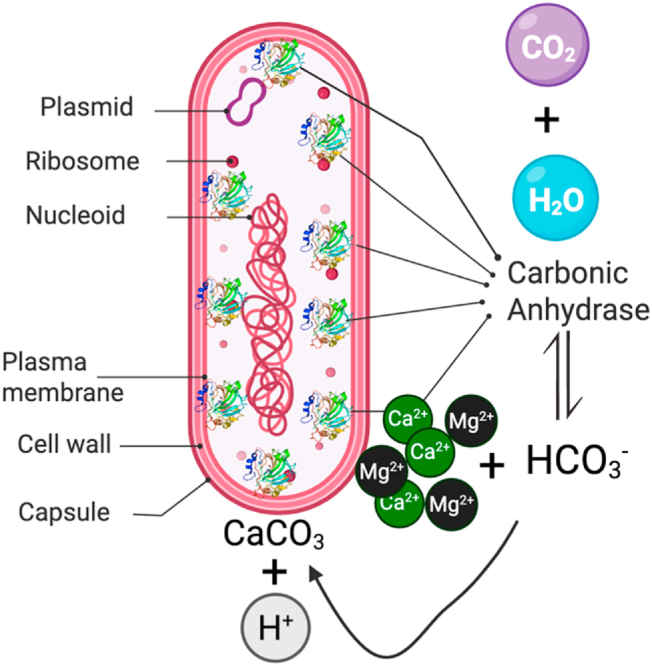
Conceptual diagram illustrating CA-mediated carbonate precipitation. CO_2_ diffuses into the extracellular space and is hydrated by surface-associated CA, forming HCO_3_^−^ and H^+^. Bicarbonate then combines with Ca^2+^ to precipitate CaCO_3_. EPS surrounding the cell may bind Mg^2+^ and Ca^2+^, enhancing mineral nucleation and favoring high-magnesium calcite (HMC) formation.

These findings suggest that biomineralization may not be a passive response to environmental conditions but could be intrinsically linked to microbial metabolic and genetic capabilities. The high CA activity observed in *V. salarius* and *V. marismortui* suggests a strong correlation between enzymatic function and carbonate precipitation. This is likely due to their ability to generate bicarbonate ions, thereby creating supersaturation conditions that are necessary for mineral formation [44]. In this study, both intracellular and extracellular fractions were evaluated for carbonic anhydrase (CA) activity using the esterase assay. No detectable activity was observed in the extracellular fractions under the assay conditions. However, scanning electron microscopy (SEM) revealed bacterial cells embedded within the mineral matrix, suggesting that carbonate precipitation occurs in close association with the cell surface. This observation implies a potential role for membrane-bound or periplasmic CA, rather than secreted enzymes, in facilitating localized supersaturation and nucleation. Future investigations employing immunolocalization or surface proteomic techniques will be necessary to confirm the spatial distribution of CA and further elucidate its functional role in microbial mineralization. This enzymatic pathway is schematically illustrated in Fig. 7 where CA catalyzes the hydration of CO_2_ into bicarbonate (HCO_3_^−^) and protons. Bicarbonate subsequently reacts with Ca^2+^ to form CaCO_3_, while EPS near the cell wall facilitates crystal stabilization, especially in the presence of Mg^2+^.

These findings align with prior research highlighting the role of bacteria in carbonate formation under hypersaline conditions [[45], [46], [47], [48], [49]]. The ability of *Virgibacillus* species to mediate carbonate precipitation without urease activity suggests a distinct biomineralization pathway, positioning them as promising candidates for both biotechnological and environmental applications. This enzymatic mechanism offers a sustainable alternative for carbon sequestration technologies, particularly in saline environments where conventional methods may be less effective [50]. The findings underscore the potential of integrating microbial biomineralization into innovative environmental technologies, contributing to global carbon management strategies [51,52]. Furthermore, the results highlight the enzymatic dependency of microbial biomineralization and indicate that *Virgibacillus salarius* and *Virgibacillus marismortui* are particularly well-adapted to carbonate precipitation under varying salinity conditions.

However, this study is limited by its focus on a select group of bacterial taxa and the constraints of short-term laboratory conditions, which may not fully capture the complexity of natural biomineralization processes. Future research incorporating metagenomic analyses, long-term in situ studies, and detailed geochemical characterizations will be essential for providing a more comprehensive understanding of microbial-driven carbonate precipitation in sabkha environments.

## 5 Conclusion

This study demonstrated that *V*. *salarius* and *V*. *marismortui* exhibit a remarkable ability to mediate carbonate mineralization across diverse media, primarily driven by their consistently high CA activity. These findings suggest that specific enzymatic and possibly genetic adaptations enable these strains to thrive under varying environmental conditions and efficiently facilitate biomineralization. In contrast, *V*. *chiguensis* and *V*. *dokdonensis* displayed limited mineral formation potential, restricted to the nutrient-rich MD1 medium, despite similar external conditions. This disparity underscores the dominance of strain-specific metabolic capabilities over environmental factors in governing biomineralization outcomes.

The ability of *V. salarius* and *V. marismortui* to form carbonate minerals in saline environments underscores both their ecological significance and their potential applications in microbial-driven carbon sequestration technologies. These findings contribute to a broader understanding of microbial roles in carbonate precipitation and reinforce the importance of sabkha ecosystems as unique blue carbon reservoirs. Moreover, the demonstrated biomineralization potential of these strains in hypersaline conditions underscores their viability in developing innovative, eco-friendly carbon capture technologies. These insights have promising biotechnological applications, particularly for microbial-based carbon capture and storage systems, while also emphasizing the need to conserve sabkha ecosystems as critical components of global carbon cycling.

## CRediT authorship contribution statement

**Khaled Naja:** Writing – original draft, Visualization, Investigation, Formal analysis. **Sara H. Alhadidi:** Writing – original draft, Investigation, Formal analysis. **Hadil Elsayed:** Writing – original draft, Investigation. **Jassim Abdulla A. Al-Khayat:** Writing – review & editing, Resources. **Fadhil Sadooni:** Writing – review & editing, Supervision, Formal analysis. **Hamad Al Saad Al-Kuwari:** Writing – review & editing, Resources, Project administration, Funding acquisition. **Zulfa Ali Al Disi:** Writing – review & editing, Visualization, Methodology, Investigation, Formal analysis, Data curation, Conceptualization.

## Declaration of generative AI and AI-assisted technologies in the writing process

During the preparation of this work, the author(s) used ChatGPT (OpenAI) for rephrasing text, improving grammar, and checking the logical flow of certain sections of the manuscript. Following this, the author(s) carefully reviewed and edited the content to ensure accuracy and take full responsibility for the final version of the manuscript**.**

## Funding

Research reported in this publication was supported by the Qatar Research Development and Innovation Council (QRDI) grants [ARG 01-0604-230489, NPRP 13S-0207-200291 and NPRP 12S-0313-190349]. The content is solely the responsipility of the authors and does not necessarily represent the official views of Qatar Research Development and Innovation.

## Declaration of competing interest

The authors declare that they have no known competing financial interests or personal relationships that could have appeared to influence the work reported in this paper.

## Data Availability

Data will be made available on request.
